# Protective Effects of *Lactobacillus*‐Derived Probiotics and Postbiotics on Renal and Reproductive Function in *Escherichia coli*–Infected Rats

**DOI:** 10.1002/vms3.71258

**Published:** 2026-09-29

**Authors:** Saeid Sedghi, Zahra Keshtmand, Saba Hosseini

**Affiliations:** ^1^ Department of Biology CT.C Islamic Azad University Tehran Iran

**Keywords:** *Escherichia coli*, gene expression, kidney, postbiotic, probiotic, testis, Wistar rat

## Abstract

**Background:**

*Escherichia coli* infections of the genitourinary tract are a major cause of renal and reproductive dysfunction in males, often impairing spermatogenesis and fertility. Probiotics and postbiotics have emerged as microbiota‑based interventions with strain‑specific immunomodulatory and antioxidant properties.

**Objectives:**

This experimental study evaluated the effects of a probiotic or postbiotic supplementation containing *Lactobacillus helveticus*, *Lactobacillus rhamnosus*, and *Lactobacillus casei* on renal and testicular tissues, as well as sperm parameters, in male Wistar rats infected with *E. coli*.

**Methods:**

Twenty‐eight male Wistar rats (200–220 g) were randomly assigned to four groups: control, *E. coli*–infected, *E. coli* + probiotic (10^9^ CFU/mL), and *E. coli* + postbiotic (10^9^ CFU/mL). Probiotic or postbiotic supplementation was administered orally (gavage) once daily for 35 consecutive days, starting immediately after the final day of bacterial challenge. Biochemical markers (urea, creatinine, sodium, potassium), histological changes, total antioxidant capacity (TAC), gene expression (IL‑6, TNF‑α, Bax, Bcl‑2), and sperm quality were assessed. Statistical analysis was performed using one‑way ANOVA with Tukey's post hoc test (*p* < 0.05).

**Results:**

*E. coli* infection significantly impaired renal and testicular histology, elevated pro‑inflammatory and pro‑apoptotic gene expression, reduced TAC, and deteriorated sperm parameters. Probiotic and postbiotic supplementation mitigated these effects, restoring antioxidant capacity, enhancing Bcl‑2 expression, reducing IL‑6 and TNF‑α levels, and improving sperm viability and morphology. Histological evaluation confirmed partial recovery of kidney and testicular structures in treated groups.

**Conclusion:**

Multi‑strain probiotic–postbiotic mixtures confer protective effects against infection‑induced oxidative stress, apoptosis, and inflammation in renal and testicular tissues, while improving sperm quality. These findings highlight their translational potential as adjunctive therapies for infection‑associated male infertility and renal impairment.

## Graphical abstract

A *Lactobacillus*‐derived probiotic and postbiotic mixture alleviates *E. coli*–induced renal and reproductive dysfunction by modulating oxidative stress, inflammatory responses, and apoptosis, leading to improved tissue integrity and sperm quality.

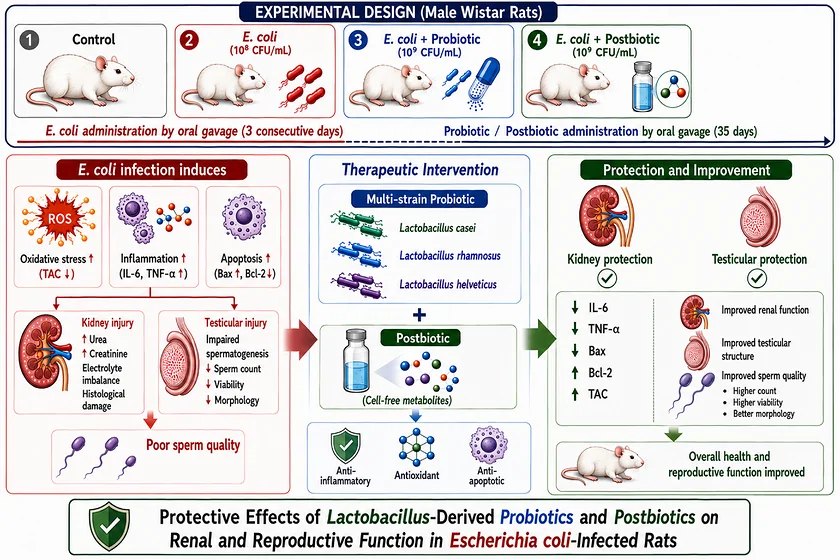

## 1 Introduction

Genitourinary tract infections represent a significant category of bacterial diseases affecting the urinary and reproductive systems. Among the causative agents, *Escherichia coli* remains the predominant pathogen. This facultative anaerobic, rod‐shaped Gram‐negative bacillus is a natural inhabitant of the intestinal microbiota in humans and animals; however, pathogenic strains are frequently implicated in gastrointestinal disturbances and extraintestinal infections (Peng et al. 2024). In the urinary tract, *E. coli* infection manifests as pyelonephritis when the upper tract is involved, or as cystitis when localized to the bladder. Urinary tract infections (UTIs) are among the most common bacterial illnesses worldwide and pose a substantial threat to adult health. In men, recurrent UTIs are closely associated with reproductive dysfunction, contributing to impaired fertility outcomes (Dolin et al. 2005; Govindarajan et al. 2024).

Male infertility is a multifactorial condition influenced by genetic predisposition, environmental exposures, and infectious insults (Komijani et al. 2018). Evidence suggests that bacterial infections, through immune‐mediated responses and apoptotic pathways, play a pivotal role in disrupting spermatogenesis and testicular function. The testes, epididymis, and accessory glands are particularly vulnerable to microbial invasion, with *E. coli* being the most frequently identified pathogen in male reproductive tract infections (Coutton et al. 2016). Clinical and experimental studies further demonstrate a strong correlation between *E. coli* infection and deterioration of sperm quality, particularly with advancing age (Kaur and Prabha 2014; Tao et al. 2017).

Although antibiotics remain the cornerstone of UTI management (Mändar et al. 2017), their long‐term efficacy is increasingly compromised by the emergence of antimicrobial resistance. Recurrent infections and reduced therapeutic responsiveness highlight the urgent need for alternative or adjunctive strategies (Souho et al. 2015; Chikindas et al. 2018).

Probiotics have emerged as promising biotherapeutic agents, capable of modulating host microbiota and conferring protective health benefits through colonization, competitive exclusion, and attenuation of pathogen virulence (Suez et al. 2018; Paone and Cani 2020). Mechanistically, probiotics reinforce mucosal barriers, inhibit pathogen adhesion, and neutralize bacterial toxins (Adamkova 2019).

Complementing these live microbial interventions, postbiotics—defined as nonviable microbial products or metabolic byproducts—exhibit diverse bioactive properties. Derived via fermentation or physicochemical processing, postbiotics encompass proteins, lipids, carbohydrates, vitamins, and metabolites such as short‑chain fatty acids. They exert immunomodulatory, antimicrobial, anti‑inflammatory, antioxidant, and anti‑proliferative effects, thereby offering therapeutic potential across diverse clinical and experimental contexts (Asefa et al. 2025; Keshtmand et al. 2025).

Clinical evidence highlights the therapeutic utility of *Lactobacillus* strains in controlling genitourinary infections. In males, *Lactobacillus* species contribute to maintaining a balanced microbiota within the urinary and reproductive tract, where their production of lactic acid, hydrogen peroxide, and antimicrobial metabolites helps suppress pathogenic colonization and modulate local immune responses (Coutton et al. 2016; Komijani et al. 2018). Recent studies demonstrate that probiotic‑derived metabolites exert antimicrobial, antioxidant, and immunomodulatory effects, reinforcing their role as adjunctive strategies in infection prevention and male reproductive health (Cannarella et al. 2021; Zhu et al. 2021).

Despite considerable progress in probiotic and postbiotic research, their functional potential in safeguarding male reproductive health remains insufficiently characterized. To date, no systematic investigations have assessed the impact of probiotic–postbiotic formulations on renal integrity, testicular function, and sperm quality under conditions of *E. coli*–induced infection. The present study seeks to bridge this critical gap by examining the therapeutic efficacy of a native Iranian probiotic and postbiotic—comprising *Lactobacillus casei*, *Lactobacillus rhamnosus*, and *Lactobacillus helveticus*—in male reproduction of Wistar rats experimentally challenged with *E. coli*. By integrating probiotic and postbiotic strategies, this work aims to generate novel insights into microbiota‐based interventions for infection‐associated male infertility, thereby advancing translational approaches to reproductive and urinary tract health.

## 2 Materials and Methods

### 2.1 Experimental Animals

Twenty‐eight adults male Wistar rats (200–250 g) were obtained from Shahid Beheshti University (Tehran, Iran). Animals were acclimatized for 1 week in the animal facility of the Faculty of Basic Sciences, Islamic Azad University, Central Tehran Branch, under controlled conditions: 12 h light/dark cycle, ambient temperature of 23 ± 2^∘^C, and ad libitum access to food and water. All procedures were performed at consistent times of day to minimize circadian variation. Body weights were recorded using a digital scale prior to surgical interventions and behavioural assessments. Animal care and experimental procedures strictly adhered to institutional guidelines for the use of laboratory animals (Mändar et al. 2017). Ethical approval was obtained under code IR.IAU.CTB.REC.1401.091.

### 2.2 Preparation of Multi‐Strain Probiotics and Postbiotics

Probiotic and postbiotic powders were purchased from Takgen Company. Postbiotics were prepared according to the manufacturer's protocol. The probiotic mixture contained *L. casei* (IBRC‐M10784), *L. rhamnosus* (IBRC‐M11322), and *L. helveticus* (TG‐35). Postbiotics were prepared by suspending 1 g of powder in 10 mL sterile distilled water, incubating at 37°C for 24 h, centrifuging at 5000 rpm for 10 min, and filtering through a 0.22‐µm membrane to obtain cell free metabolites. Probiotics were reconstituted in sterile saline (1 g in 10 mL), vortexed thoroughly, and adjusted to 1 × 10^9^ CFU/mL using McFarland standards.

#### 2.2.1 Bacterial Culture

Pathogenic *E. coli* strains were obtained from the Genetic Resources Center of Iran. Cultures were grown on Eosin Methylene Blue (EMB) and MacConkey agar (Merck, Germany) at 37°C for 24 h. Seven *E. coli* colonies (two lactose‑negative and five lactose‑positive) that were biochemically characterized using TSI, Simon Citrate, MR‑VP, and EMB/MacConkey morphology were pooled and used as the challenge inoculum. Phenotypic identification was selected because the aim of this study was to confirm pathogenic characteristics relevant to infection induction rather than molecular strain typing. For infection preparation, bacteria were cultured in tryptic soy broth (TSB) at 37°C for 24 h, centrifuged at 2000 rpm for 20 min (Behdad, Iran), washed with 0.5× phosphate‐buffered saline (PBS), and resuspended (Peng et al. 2024). Optical density was adjusted to OD630 = 0.5 using a spectrophotometer (Thermo Scientific, USA), corresponding to 1 × 10^8^ CFU/mL. Each rat received 1 mL of bacterial suspension orally for 3 consecutive days.

### 2.3 Experimental Design

A total of 28 Wistar male rats, which were kept under the mentioned conditions, were distributed into four groups: (1) the control group, (2) infected with *E. coli* (10^8^ CFU/mL), (3) the infected model + receiving postbiotic (10^9^ CFU/mL), and (4) infected model + receiving probiotic (10^9^ CFU/mL). Body weight was recorded on days 0, 18, and 35 of the experiment. Probiotic or postbiotic supplementation was administered orally (gavage) once daily for 35 consecutive days, starting immediately after the final day of bacterial challenge. At the end of the experimental period, testicular weight was measured using a digital scale, and testicular volume was assessed using callipers to ensure accurate morphometric evaluation.

#### 2.3.1 Biochemical Study

At the end of the study, rats were anesthetized with ketamine (87 mg/kg) and xylazine (13 mg/kg). Blood samples were collected via cardiac puncture, and kidneys and testes were excised for histological, molecular, and antioxidant analyses. Serum was separated by centrifugation at 4000 rpm for 10 min at room temperature (Komijani et al. 2018). Biochemical parameters (creatinine, sodium, potassium, and urea) were measured using commercial assay kits (Pars Azmoon, Iran) and analyzed with an autoanalyzer (Hitachi, Japan).

### 2.4 Examination of Kidney and Testicle Histology

Kidney and testis tissues were collected immediately after euthanasia. Tissue fragments (3–5 mm) were fixed in 4% paraformaldehyde for 24 h, dehydrated through graded ethanol, cleared in xylene, and embedded in paraffin. Paraffin blocks were sectioned at 5‐µm thickness using a rotary microtome. Routine haematoxylin and eosin (H&E) staining was performed for general histological evaluation, and periodic acid–Schiff (PAS) staining was applied to kidney sections to assess basement membrane integrity (Li et al. 2006). Images were captured at 100× magnification using an automated slide‑scanning system (Axio Scan Z1; Zeiss Korea, Seoul, Korea). Histological evaluation was performed independently by three blinded pathologists, and inter‑observer agreement was assessed (*κ* = 0.82), confirming high reliability of scoring.

### 2.5 Sperm Extraction

Fragments of the epididymis were placed in a plate containing sperm‐specific culture medium and distilled water, then mechanically minced. After complete homogenization, the suspension was incubated for 20 min. The sperm solution was transferred onto a slide and stained with Papanicolaou and Eosin Y dyes (Merck, Germany). Finally, a neobar slide was used to count spermatozoa (Coutton et al. 2016).

### 2.6 Measurement of the Total Antioxidant Capacity

Total Antioxidant Capacity (TAC) levels in kidney and intestinal tissues were measured using a TAC kit (Naxifer, Iran). Approximately 50–100 mg of tissue was weighed, and lysing buffer was added at a volume 10 times the tissue weight. Samples were homogenized and centrifuged at 10,000 rpm for 10 min, after which the supernatants were collected for analysis. Subsequent steps were performed according to the kit protocol. A standard sample was included alongside the test samples. Optical absorption was measured at 593 nm as the final step (RahimBakhsh et al. 2025).

### 2.7 Investigation of Gene Expression by Real‐Time Polymerase Chain Reaction Method

The expression of apoptotic and inflammatory genes was quantified by real‐time polymerase chain reaction (PCR). Total RNA was extracted using RNX‐plus reagent (SinaClon, Iran). cDNA synthesis was performed from 1000 ng RNA using the RevertAid First Strand cDNA Synthesis Kit (Fermentas, USA). PCR reactions were conducted in duplicate in 25 µL volumes with SYBR Green Master Mix (Amplicon, Denmark). GAPDH served as the internal control. Relative gene expression was calculated using the ΔΔCt method with the REST software (Tao et al. 2017). Primer sequences are listed in Table 1. Primer sequences were designed using standard primer design software and validated through NCBI Primer‑BLAST to ensure specificity and absence of off‑target amplification.

**TABLE 1:** Experimental primer sequences.

Gene	Primer sequence
TNF‐α	Forward: 5’‐ACTGAACTTCGGGGTGATTG‐3 Reverse: 5’‐GCTTGGTGGTTTGCTACGAC‐3
IL‐6	Forward: 5'‐TGATGGATGCTTCCAAACTG‐3 Reverse: 5'‐GAGCATTGGAAGTTGGGG TA‐3
BAX	Forward: 5'‐CGGCGAATTGGAGATGAACTGG‐3 Reverse: 5'‐CTAGCAAAGTAGAAGAGGGCAACC‐3
BCL2	Forward: 5'‐TGTGGATGACTGACTACCTGAACC‐3 Reverse: 5'‐CAGCCAGGAGAAATCAAACAGAGG‐3
GAPDH	Forward: 5'‐AAGATCCTGACCGAGCGTGG‐3 Reverse: 5'‐CAGCACTGTGTTGGCATAGAGG‐3

### 2.8 Statistical Analysis

All statistical analyses were performed using SPSS version 23 (IBM Corp., Armonk, NY, USA). Data were expressed as mean ± standard deviation (SD). Group comparisons were conducted using one‐way analysis of variance (ANOVA). When significant differences were detected, Tukey's honest significant difference (HSD) post hoc test was applied to evaluate pairwise differences in biochemical parameters, histological scores, sperm characteristics, antioxidant capacity, and gene expression levels. A threshold of *p* < 0.05 was considered statistically significant.

## 3 Results

### 3.1 Biochemical Identification of *E. coli*

Biochemical characterization confirmed the identity of the bacterial isolate as *E. coli*. In triple sugar iron (TSI) medium, glucose fermentation and gas production were positive, whereas hydrogen sulphide production was absent. Simon citrate and Voges–Proskauer (VP) tests were negative, while the Methyl Red (MR) test was positive. Collectively, these results verified the presence of *E. coli* in the cultures.

### 3.2 Histological Findings in Kidney Tissue

Histopathological evaluation of kidney sections stained with H&E (Figures 1 and 2; Table 2) revealed distinct differences among the experimental groups. In the control group (Figure 1B), renal architecture was preserved, with intact glomeruli; normal tubular epithelium; and absence of vacuolar degeneration, haemorrhage, or necrosis. In contrast, the *E. coli*–infected group (Figure 1A) exhibited marked pathological changes, including vacuolar degeneration, extensive haemorrhage, collagen fibre deposition, cellular necrosis, and pronounced lymphocyte infiltration. These alterations were consistent with severe infection‑induced renal injury. Treatment with probiotics (Figure 1C) or postbiotics (Figure 1D) attenuated these pathological changes. Both groups demonstrated partial restoration of renal histology, with reduced vacuolization, diminished inflammatory infiltration, and improved glomerular structure compared with the infected group. Although tissue damage was not completely resolved, probiotic and postbiotic supplementation clearly promoted histological recovery.

**FIGURE 1:**
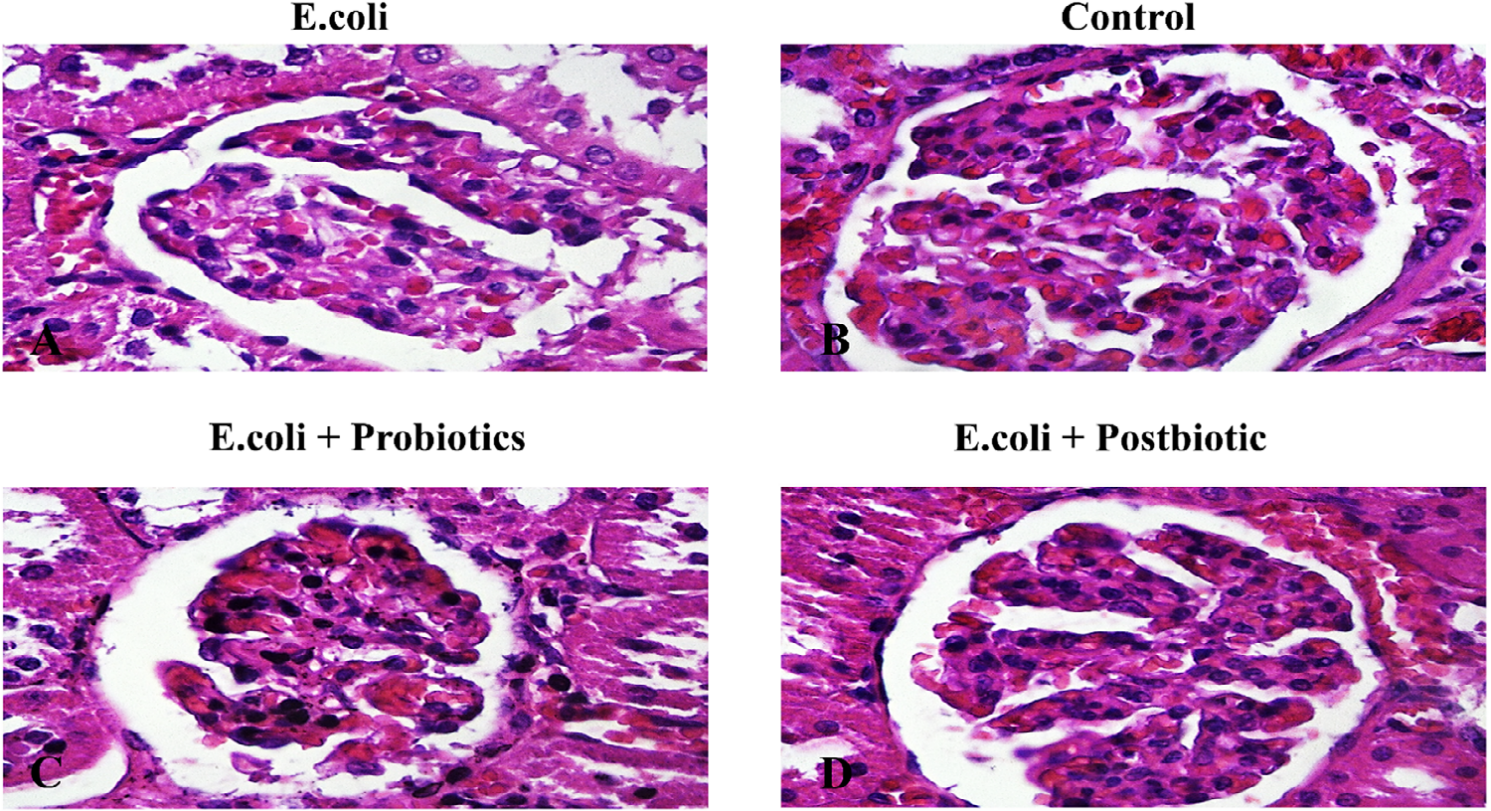
Histopathological photomicrographs of kidney tissue in different groups at the end of the experiment: (A) *E. coli*–infected group, (B) control, (C) *E. coli*–infected rats treated with probiotics, and (D) *E. coli*–infected rats treated with postbiotics. Sections were stained with haematoxylin and eosin (H&E); magnification ×100.

**FIGURE 2:**
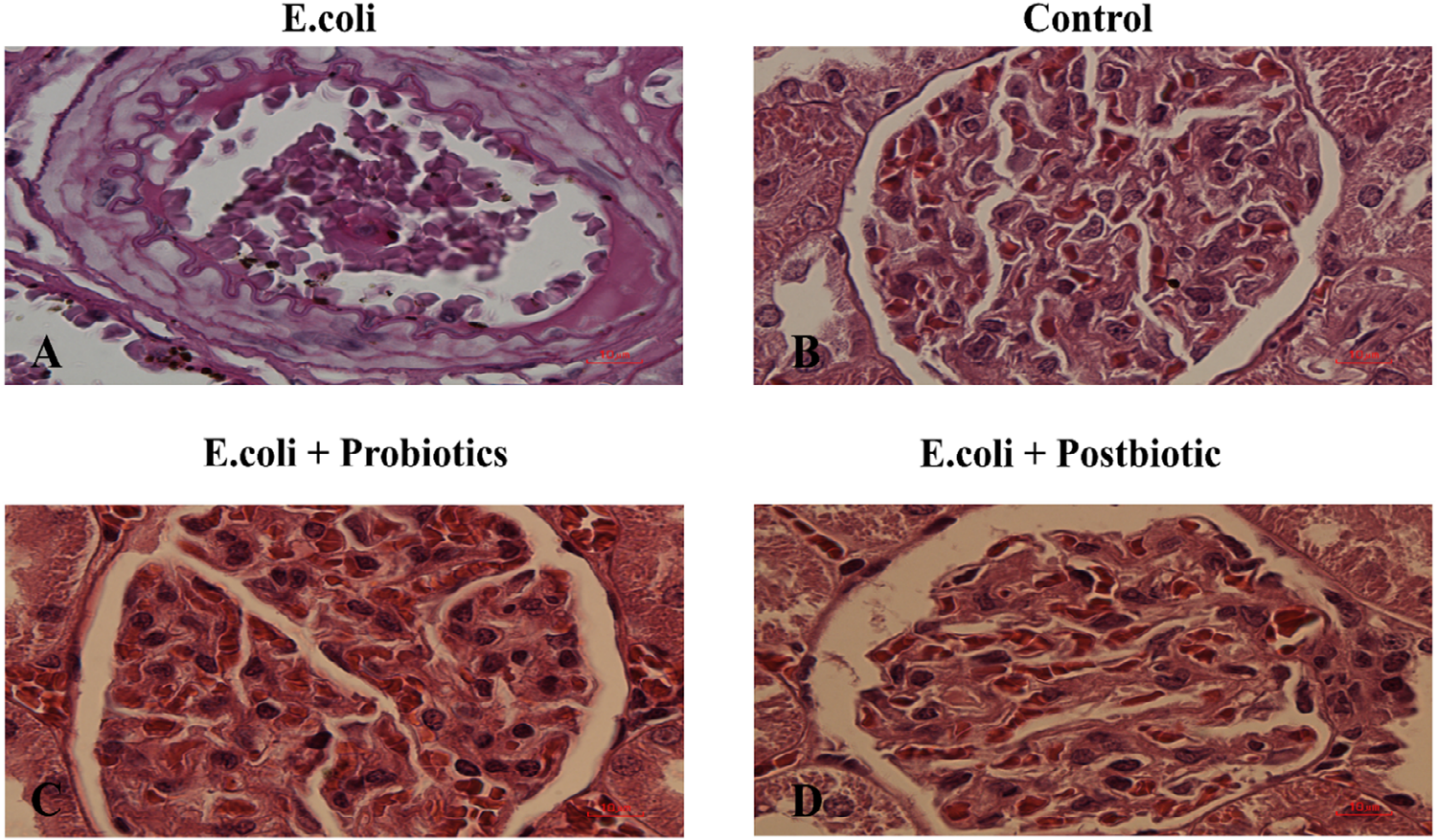
Histopathological photomicrographs of kidney tissue in different groups at the end of the experiment; (A) *E. coli*‑infected group, (B) control group, (C) *E. coli*‑infected rats treated with probiotics, and (D) *E. coli*‑infected rats treated with postbiotics. Sections were stained with periodic acid–Schiff (PAS); magnification ×100.

**TABLE 2:** Kidney parameters in different groups.

Parameters	Control	*E. coli* (10^8^ CFU/mL)	*E. coli* (10^8^ CFU/mL) + probiotics 10^9^ (CFU/mL)	*E. coli* (10^8^ CFU/mL) + postbiotic 10^9^ (CFU/mL)
Vacuolar degeneration‐type changes	−	+++	++	++
Bleeding in the kidney tissue	−	++++	++	++
Glomerular dilation	−	+++	+	+++
Collagen fibre accumulation	−	+++	+	++
Cellular necrosis	−	+++	++	+++
Apoptotic parietal epithelial cells (Bowman's capsule)	−	+++	+	+++
Cytoplasmic vacuolization	−	++++	++	++
Interstitial lymphocyte infiltration	−	++++	++	++

*Note*: –: normal; +: minimal; ++: mild; +++: moderate; ++++: severe.

### 3.3 Histological Findings in Testicular Tissue

Histological evaluation of testicular sections stained with H&E (Figure 3; Table 3) revealed marked differences among the experimental groups. In the control group (Figure 3B), seminiferous tubules exhibited normal morphology with intact germinal epithelium, well‑organized cell layers, preserved nuclei and cytoplasm, and regular lumen diameter, indicating healthy testicular architecture. In contrast, the *E. coli*–infected group (Figure 3A) displayed severe pathological alterations, including disruption of the germinal epithelium, accumulation of inflammatory cells, reduction in seminiferous tubule size, and destruction of luminal structure. These findings confirm infection‑induced testicular damage and impaired spermatogenic integrity. Treatment with probiotics (Figure 3C) or postbiotics (Figure 3D) attenuated these pathological changes. Both groups demonstrated partial restoration of seminiferous tubule morphology, reduced inflammatory infiltration, and improved germinal epithelium organization compared with the infected group. Although complete recovery was not achieved, probiotic and postbiotic supplementation clearly promoted histological repair and preservation of testicular tissue integrity.

**FIGURE 3:**
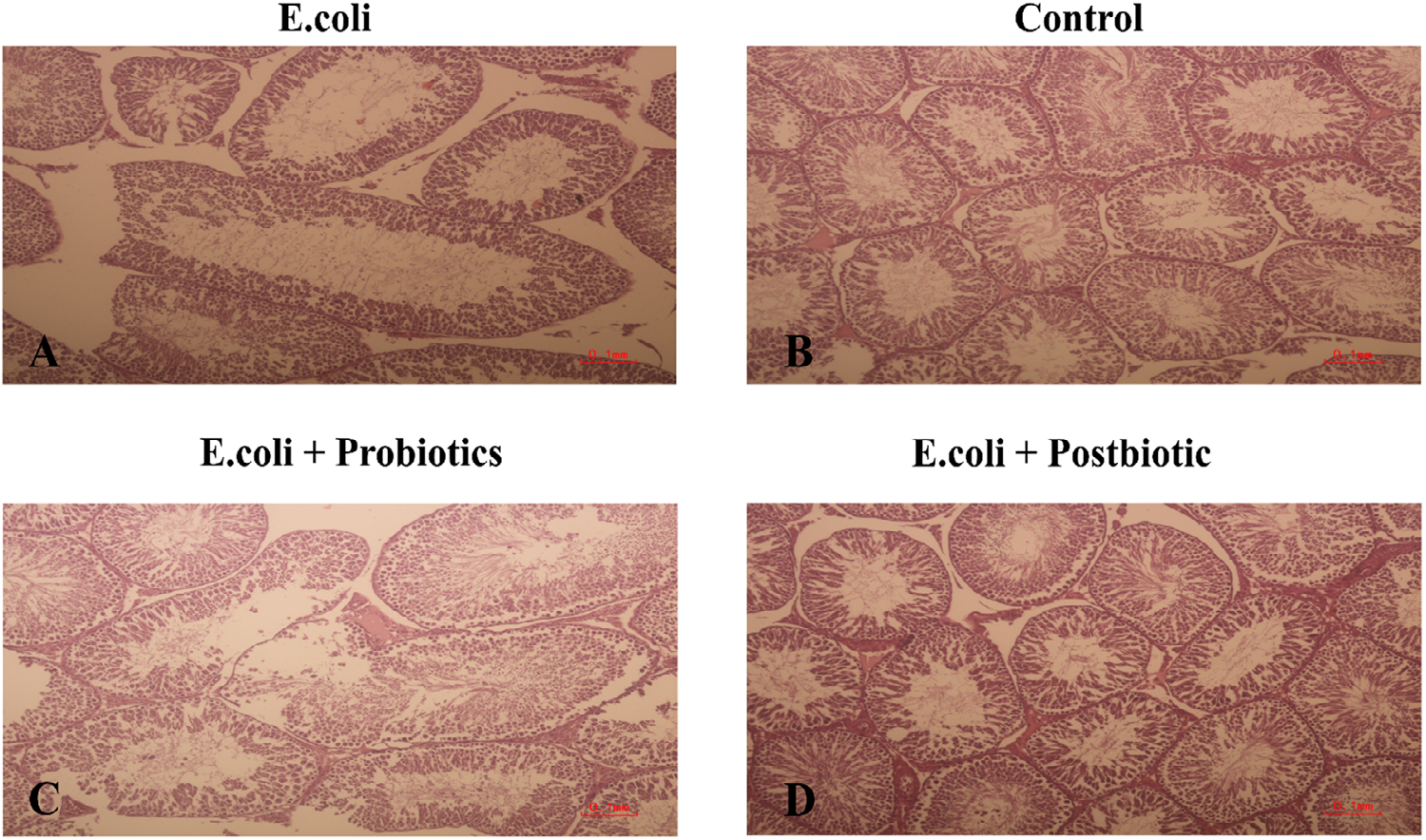
Histological testicular tissue changes in different groups. (A) *E. coli*‑infected group, (B) control group, (C) *E. coli*‑infected rats treated with probiotics, and (D) *E. coli*‑infected rats treated with postbiotics. Sections were stained with haematoxylin and eosin (H&E); magnification ×100.

**TABLE 3:** Comparison of testicular tissue in different groups.

Different groups	Testicle weight	Testicular volume	Diameter of seminiferous tubules (µm)	Lumen diameter (μ)	Cell layer thickness (μ)
Control	1.45 ± 0.051	1.950 ± 0.079	243.27 ± 6.24	510.2 ± 15.2	296 ± 7.9
*E. coli* receiving	1.20 ± 0.076^***^	1.35 ± 0.12^***^	173.13 ± 6.19^***^	667.6 ± 12.6^***^	149.4 ± 5.6^***^
*E. coli* receiving group + probiotics 10^9^(CFU/mL)	1.32 ± 0.54^*^	1.58 ± 0.046^***^	214.05 ± 4.38^[Link]^, ^[Link]^	532 ± 10.86^[Link]^, ^[Link]^	244.3 ± 8.7^[Link]^, ^[Link]^
*E. coli* receiving group + postbiotic 10^9^(CFU/mL)	1.39 ± 0.64^*^	1.6 ± 0.032^***^	212 ± 3.98^[Link]^, ^[Link]^	537 ± 9.53^[Link]^, ^[Link]^	250.1 ± 9.1^[Link]^, ^[Link]^

*Note*: Data are presented as mean ± SD; ANOVA tests were used for analysis.

Comparison with the control group:

^*^ *p* < 0.05.

^**^ *p* < 0.01.

^***^ *p* < 0.001.

Comparison with the infected group:

^#^ *p* < 0.05.

^##^ *p* < 0.01.

In Table 3, the decrease in testicular weight, testicular volume, and seminiferous tubule diameter in the *E. coli*–infected group compared to the control group was statistically significant. All measurements were performed at the end of the experimental period using callipers. In contrast, lumen diameter and germ cell layer thickness showed a significant increase in the infected group relative to controls. Probiotic and postbiotic treatments partially restored these parameters toward normal values, indicating their protective effects against *E. coli*–induced testicular injury.

### 3.4 Chemical Serum Factors Result

As shown in Figure 4A, serum urea levels were significantly elevated in the *E. coli*–infected group compared with the control group (*p* < 0.001). Similarly, rats receiving *E. coli* in combination with probiotics and postbiotics exhibited higher urea levels than controls (*p* < 0.01). However, probiotic–postbiotic treatment significantly reduced urea concentrations compared with the *E. coli*‐only group (*p* < 0.05).

**FIGURE 4:**
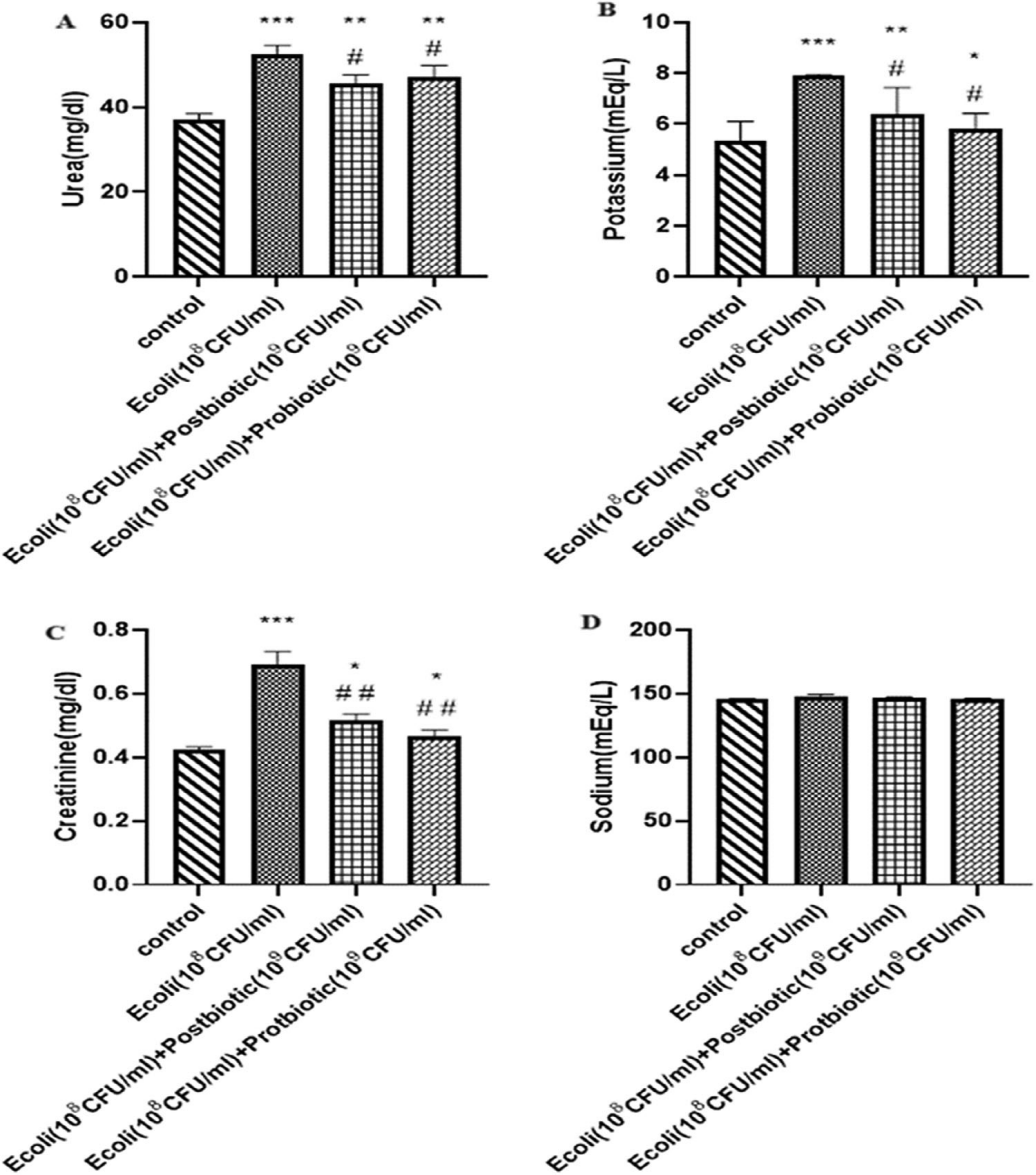
The levels of chemical serum factors (urea, potassium, creatinine, and sodium) alteration in male Wistar rats in (*E. coli* + probiotic) and (*E. coli* + postbiotic) compared to *E.coli* and control group. *: comparison with the control group, *p < 0.05, **p < 0.01, ***p < 0.001. #: comparison with the infected group, # *p* < 0.05, ## *p* < 0.01.

Potassium levels followed a comparable trend (Figure 4B), with significant increases in the *E. coli*–infected and treated groups relative to controls, though the magnitude of change differed across groups (varying *p*‐values).

As illustrated in Figure 4C, serum creatinine concentrations were markedly elevated in the *E. coli*–infected group (*p* < 0.001) and in the *E. coli* + probiotic–postbiotic group (*p* < 0.05) compared with controls. Importantly, probiotic–postbiotic supplementation significantly reduced creatinine levels relative to the *E. coli*‐only group (*p* < 0.01). In contrast, sodium concentrations did not differ significantly among any of the experimental groups (Figure 4D), indicating that infection and treatment did not alter serum sodium homeostasis.

### 3.5 Total Antioxidant Capacity Results

As shown in Figure 5A, TAC levels in kidney tissue were significantly reduced in the *E. coli*–infected group compared with the control group (*p* < 0.001). Animals receiving *E. coli* in combination with probiotics and postbiotics also exhibited a significant reduction in TAC relative to controls (*p* < 0.05). Interestingly, treatment with postbiotics alone resulted in a modest increase in TAC compared with controls, although this difference did not reach statistical significance (*p* > 0.05). Importantly, probiotic–postbiotic supplementation significantly improved TAC levels compared with the *E. coli*‐only group (*p* < 0.001), indicating a restorative effect of the intervention. These findings were consistent with TAC measurements in testicular tissue (Figure 5B), where *E. coli* infection markedly reduced antioxidant capacity, and probiotic–postbiotic treatment partially restored TAC values toward control level.

**FIGURE 5:**
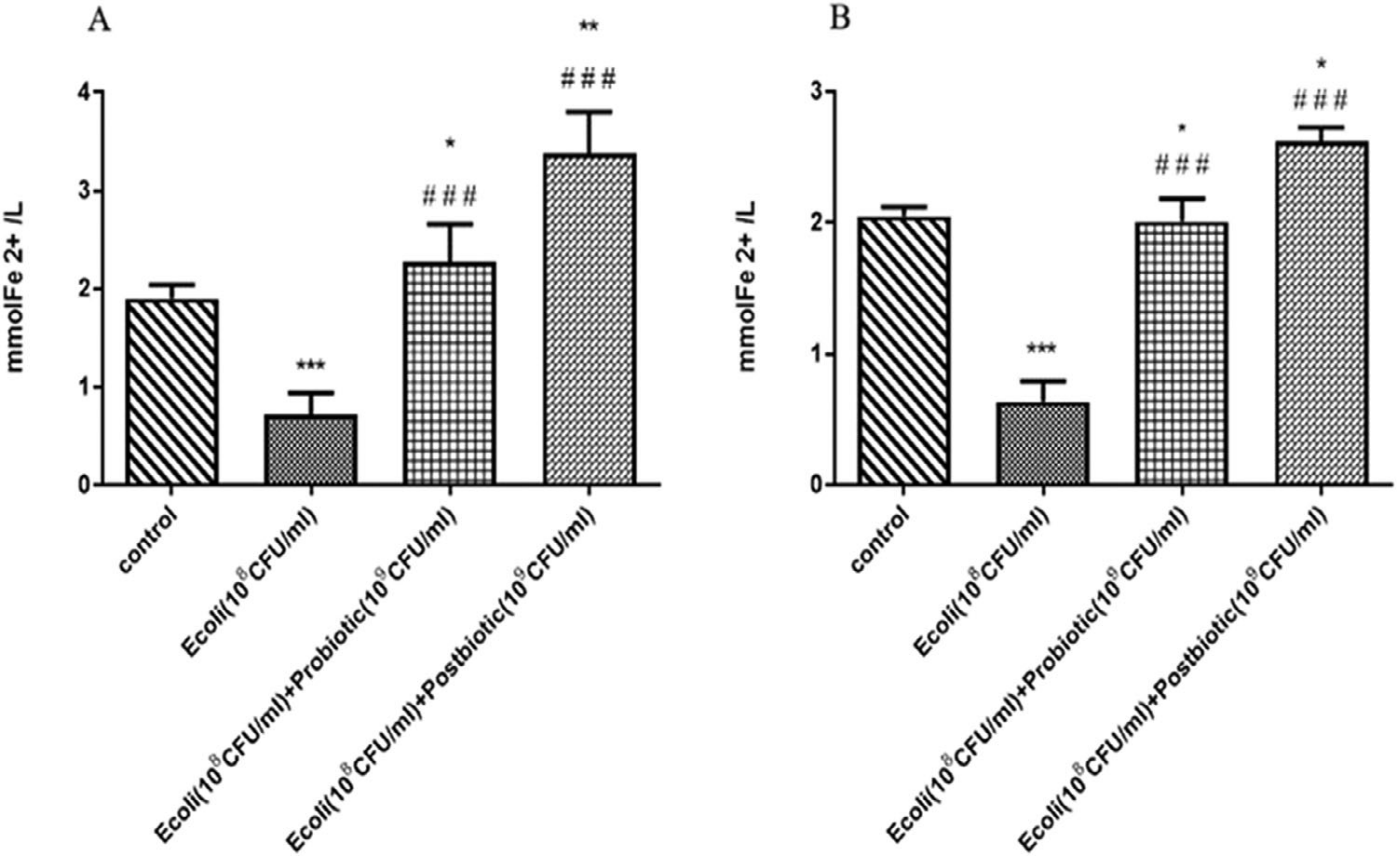
The levels of TAC (kidney (A) and testis (B)) alteration in male Wistar rats in (*E. coli* + probiotic) and (*E. coli* + postbiotic) compared to *E.coli* and control group. The mean ± SD of three experiments is used to express the data. *: comparison with the control group, *p < 0.05, **p < 0.01, ***p < 0.001. #: comparison with the infected group, ###*p* < 0.001.

### 3.6 Kidney and Testis Apoptotic Gene Expression

As shown in Figure 6A, Bax gene expression in kidney tissue was significantly elevated in the *E. coli*–infected group compared with the control group (*p* < 0.001). Both probiotic‑treated and postbiotic‑treated groups also showed increased Bax expression relative to controls (*p* < 0.05). However, treatment with either probiotic or postbiotic markedly reduced Bax expression compared with the *E. coli*‑only group (*p* < 0.001), indicating a protective effect against infection‑induced apoptosis. Consistent findings were observed in testicular tissue (Figure 6B), where Bax expression followed a similar pattern with minor variations in statistical significance. Analysis of Bcl‐2 gene expression revealed an opposite trend. In kidney tissue (Figure 6C), Bcl‐2 expression was significantly upregulated in the *E. coli*–infected group (*p* < 0.01) and further elevated in the probiotic–postbiotic treatment group (*p* < 0.001) compared with controls. Importantly, probiotic–postbiotic supplementation significantly increased Bcl‐2 expression relative to the *E. coli*‐only group (*p* < 0.01), suggesting enhanced anti‐apoptotic activity.

**FIGURE 6:**
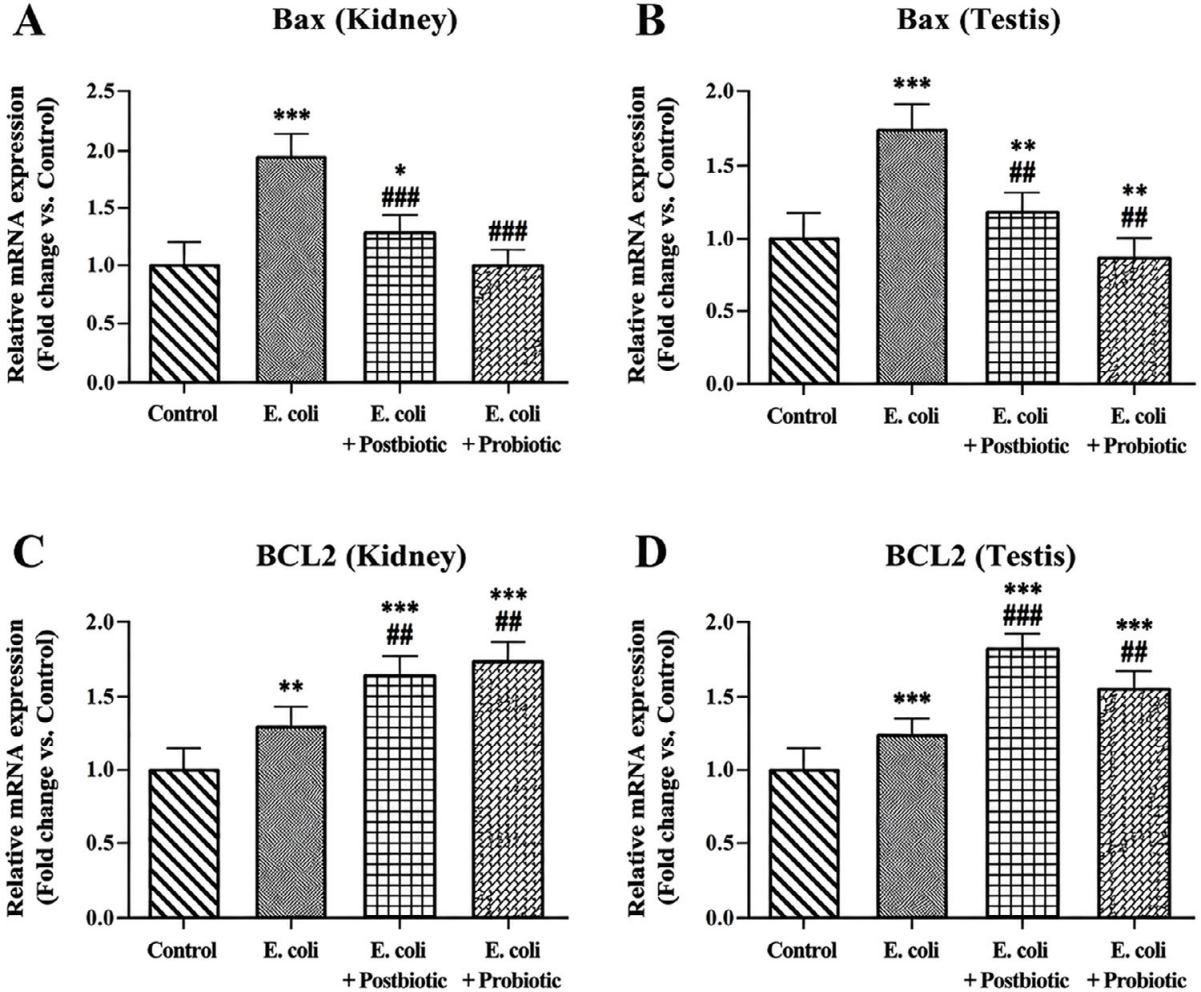
Relative mRNA expression levels of apoptosis‐related genes Bax and BCL2 in kidney and testis tissues of male Wistar rats. The expression levels of Bax (A, B) and BCL2 (C, D) were evaluated in kidney (A, C) and testis (B, D) tissues of *E. coli*–infected rats treated with probiotic and postbiotic preparations. Data are presented as fold change relative to the control group (set as 1) using the ΔΔCt normalization approach. Values are expressed as mean ± SD of three independent experiments. Statistical significance was determined by comparison with the control group (*p < 0.05, **p < 0.01, ***p < 0.001) and the *E. coli*–infected group (#*p* < 0.05, ##*p* < 0.01, ###*p* < 0.001).

Similar results were obtained in testicular tissue (Figure 6D). *Escherichia coli* infection, as well as probiotic and postbiotic treatments, significantly increased Bcl‐2 expression compared with controls (*p* < 0.001). Moreover, Bcl‐2 levels were further elevated in the probiotic (*p* < 0.01) and postbiotic (*p* < 0.001) groups compared with the infected group, reinforcing the protective role of these interventions in maintaining testicular integrity.

### 3.7 Kidney and Testis Inflammatory Gene Expression

Analysis of interleukin‐6 (IL‐6) expression in kidney tissue (Figure 7A) revealed a significant upregulation in the *E. coli–*infected group compared with controls (*p* < 0.01). In contrast, probiotic‐ and postbiotic‐treated groups did not show significant increases relative to controls. Notably, combined probiotic or postbiotic supplementation significantly reduced IL‐6 expression compared with the *E. coli*‐only group (*p* < 0.001), indicating a strong anti‐inflammatory effect.

**FIGURE 7:**
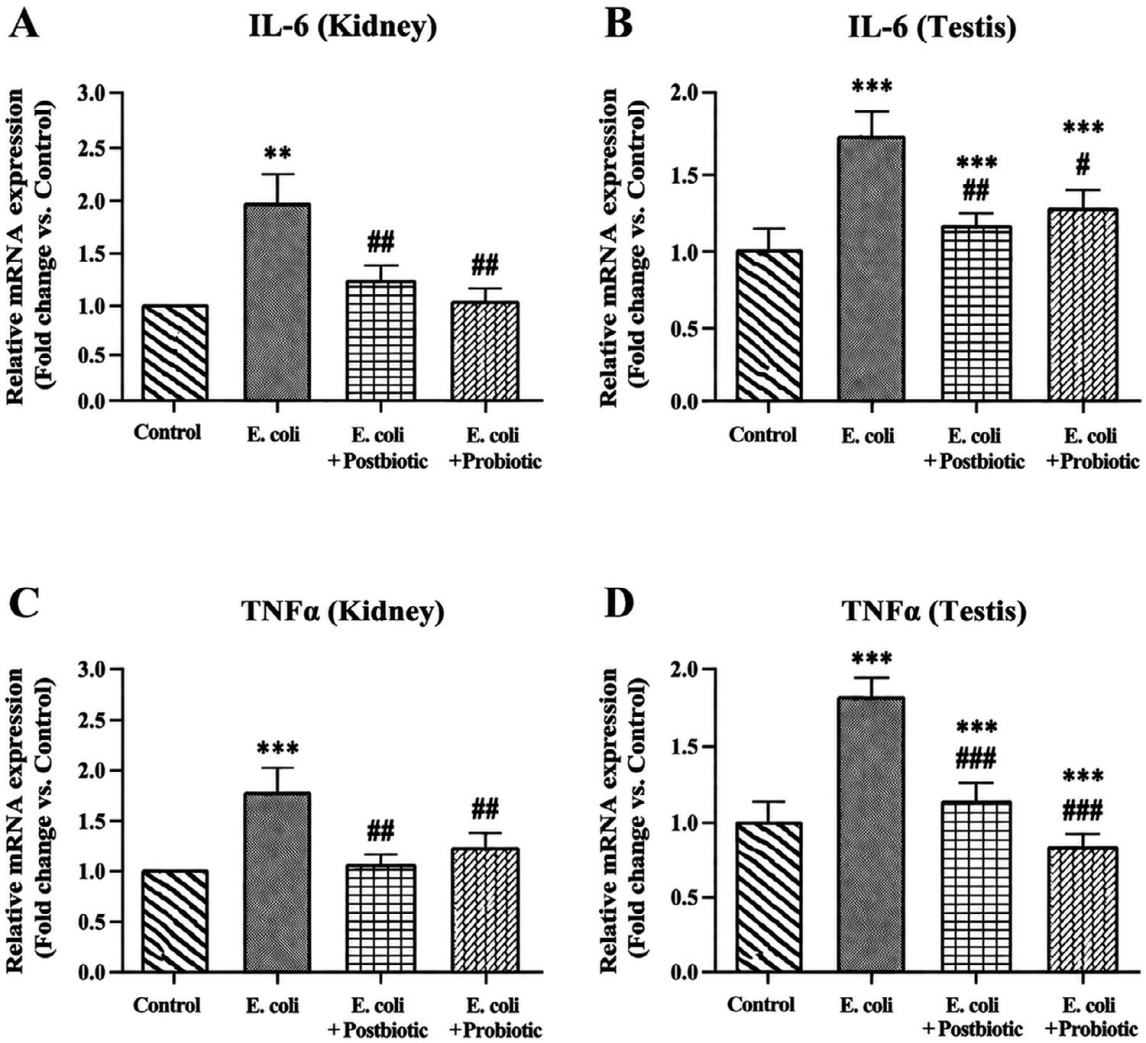
Relative mRNA expression levels of inflammatory genes IL‐6 and TNFα in kidney and testis tissues of male Wistar rats. The expression levels of IL‐6 (A, B) and TNFα (C, D) were evaluated in kidney (A, C) and testis (B, D) tissues of E. coli–infected rats treated with probiotic and postbiotic preparations. Data are presented as fold change relative to the control group (set as 1) using the ΔΔCt normalization approach. Values are expressed as mean ± SD of three independent experiments. Statistical significance was determined by comparison with the control group (*p < 0.05, **p < 0.01, ***p < 0.001) and the E. coli–infected group (#*p* < 0.05, ##*p* < 0.01, ###*p* < 0.001).

In testicular tissue (Figure 7B), IL‐6 expression was markedly elevated in the *E. coli*–infected group (*p* < 0.001). Probiotic (*p* < 0.05) and postbiotic (*p* < 0.01) treatments significantly decreased IL‐6 levels compared with the infected group, suggesting partial restoration of inflammatory balance. Tumour necrosis factor‐alpha (TNF‐α) expression in kidney tissue (Figure 7C) was significantly increased in the *E. coli*–infected group compared with the controls (*p* < 0.001). However, probiotic and postbiotic treatments did not differ significantly from controls. Importantly, TNF‐α expression was significantly reduced in both treatment groups compared with the *E. coli*‐only group (*p* < 0.01), further supporting their anti‐inflammatory role. In testicular tissue (Figure 7D), TNF‐α expression was strongly elevated in the *E. coli*–infected group relative to controls (*p* < 0.001). Probiotic and postbiotic treatments also showed increased TNF‐α levels compared with controls (*p* < 0.001), but combined probiotic–postbiotic supplementation significantly reduced TNF‐α expression compared with the infected group (*p* < 0.001).

### 3.8 Sperm Analysis

As summarized in Tables 4, 5, 6, sperm parameters were markedly affected by *E. coli* infection. In the control group, testicular tissue exhibited normal spermatogenesis, with intact sperm number, viability, and morphology, indicating healthy reproductive function. In contrast, the *E. coli*‐infected group demonstrated significant impairments, including reduced spermatogenesis, decreased sperm viability, and abnormal morphology, consistent with infection‐induced reproductive damage. Notably, rats treated with probiotics or postbiotics in conjunction with *E. coli* infection exhibited partial restoration of sperm quality. Both treatment groups showed improvements in spermatogenesis, viability, and morphology compared with the *E. coli*‐only group, suggesting that probiotic and postbiotic supplementation mitigated infection‐related damage and promoted relative repair of testicular function.

**TABLE 4:** Comparison of sperm parameters in different groups.

Sperm parameters	Control	*E. coli* (10^8^ CFU/mL)	*E. coli* (10^8^ CFU/mL) + Probiotic 10^9^(CFU/mL)	*E. coli* (10^8^ CFU/mL) + Postbiotic 10^9^(CFU/mL)
Sperm count	−	++++	++	++
Sperm viability	−	++++	+++	+++
Sperm morphology	−	++++	++	++

*Note*: –: normal; +: minimal; ++: mild; +++: moderate; ++++: severe.

**TABLE 5:** The number of spermatogonial cells, spermatocytes, and spermatids in different groups.

Sperm parameters	*E. coli* (10^8^ CFU/mL) + postbiotic 10^9^ (CFU/mL)	*E. coli* (10^8^ CFU/ml) + probiotic 10^9^ (CFU/mL)	*E. coli* (10^8^ CFU/mL)	Control
Spermatogonia (count)	81.14 ± 0.3	61.28 ± 3.11^**^	72.72 ± 3.09^[Link]^, ^[Link]^	75.24 ± 3.18^[Link]^, ^[Link]^
Spermatocyte (count)	66.43 ± 2.39	57.23 ± 4.56^**^	60.38 ± 3.80^[Link]^, ^[Link]^	61.35 ± 3.91^[Link]^, ^[Link]^
Spermatid (count)	128.58 ± 1.49	103.23 ± 3.95^**^	117 ± 1.58^[Link]^, ^[Link]^	116.06 ± 1.12^[Link]^, ^[Link]^

*Note*: Data are presented as mean ± SD; ANOVA tests were used for analysis.

compared to control group:

^*^ *p* < 0.05,

^**^ *p* < 0.01.

compared to *E.coli* group:

^#^ *p* < 0.05.

**TABLE 6:** Comparative analysis of sperm alternation in various groups of Wistar rats.

Sperm parameters	Control	*E. coli* (10^8^ CFU/mL)	*E. coli* (10^8^ CFU/mL) + probiotic 10^9^ (CFU/mL)	*E. coli* (10^8^ CFU/mL) + postbiotic 10^9^ (CFU/mL)
Spermatogonia count	−	++++	++	++
Primary spermatocyte count	−	+++++	+++	+++
Secondary spermatocyte count	−	++++	++	++
Sperm	−	++++	++	++
Diameter of seminiferous tubules	−	+++	+	+

*Note*: –: normal; +: minimal; ++: mild; +++: moderate; ++++: severe.

In addition, the number of spermatogonia, primary and secondary spermatocytes, and diameter of seminiferous tubules remained unchanged in the control group, while in the group infected with bacteria, the most damage was observed in each of these parameters.

## 4 Discussion

The global escalation of antibiotic resistance has transformed bacterial infections into a critical challenge for modern medicine. Conventional antibiotic therapies, while effective in acute management, often disrupt commensal microbial communities, thereby facilitating the persistence of resistant strains. Moreover, systemic dissemination of pathogens such as *E. coli* can lead to septicaemia, significantly increasing morbidity and mortality rates (Gutiérrez et al. 2016). Virulence factors including toxins, capsules, and pili genes have been implicated in the pathogenesis of urinary tract infections (Çiftci and Tuna 2021), underscoring the need for alternative therapeutic strategies that both suppress pathogenic activity and preserve host microbial balance.

In this study, we demonstrated that *E. coli* infection in male Wistar rats induced profound renal and testicular damage, accompanied by biochemical alterations, oxidative stress, and dysregulation of apoptotic and inflammatory gene expression. Specifically, infection elevated serum urea and creatinine, reduced total antioxidant capacity (TAC), and triggered histological degeneration in kidney and testis tissues. At the molecular level, *E. coli* infection upregulated pro‐apoptotic Bax and pro‐inflammatory cytokines (TNF‐α, IL‐6), while downregulating the anti‐apoptotic gene Bcl‐2, consistent with infection‐induced apoptosis and inflammation.

Importantly, supplementation with a native probiotic–postbiotic mixture (*L. casei*, *L. rhamnosus*, and *L. helveticus*) mitigated these deleterious effects (Keshtmand et al. 2025). Both probiotics and postbiotics improved TAC, reduced serum biochemical markers, and partially restored tissue architecture. Furthermore, treatment downregulated Bax, TNF‐α, and IL‐6, while upregulating Bcl‐2, suggesting a dual role in modulating apoptosis and inflammation. These findings highlight the therapeutic potential of microbiota‐based interventions in protecting renal and reproductive function against *E. coli*–induced injury (Zhang et al. 2022).

Our results align with previous studies demonstrating the beneficial effects of *Lactobacillus* strains on reproductive and systemic health. Moreover, Dardmeh et al. (2017) revealed beneficial benefits on male reproductive hormone levels and testicular tissue after feeding hypercholesterolemic male rats a diet supplemented with *Lactobacillus acidophilus*. According to Vílchez et al. (2015), Anguilla's spermatogenesis processes improved after receiving *L. rhamnosus* treatment for 2 weeks, as shown by transcriptome analysis and sperm parameter evaluation (Vílchez et al. 2015). Duarte et al. (2020) demonstrated that synbiotic supplementation improved growth performance and reduced oxidative stress and diarrhoea in piglets challenged with enterotoxigenic *E. coli* (Duarte et al. 2020). Similarly, previous studies reported that probiotic mixtures significantly reduced oxidative stress and inflammatory cytokines, including IL‐6, in both gastrointestinal and systemic disease models (Cannarella et al. 2021; Zhu et al. 2021; Chen et al. 2025).

Mechanistically, probiotics and postbiotics exert protective effects through multiple pathways. These include competitive exclusion of pathogens, production of antimicrobial compounds (bacteriocins, organic acids), reinforcement of epithelial barrier integrity, and modulation of host immune responses (Monteagudo‐Mera et al. 2019; Teame et al. 2020). Probiotics also regulate toll‐like receptor (TLR) signalling, dendritic cell activity, and cytokine production, thereby balancing innate and adaptive immunity (Mazziotta et al. 2023; Liu et al. 2023). The synergistic use of multiple strains, as in our study, may amplify these effects by combining diverse metabolic and immunomodulatory properties (C. Lee, Kim, et al. 2024) (Wang et al. 2020). After an 8‐week treatment with a probiotic mixture containing *Lactobacillus salivarius*, *L. casei*, and *Bifidobacterium bifidum*, the study reported a significant reduction in serum IL‐6 and other pro‐inflammatory cytokines (Wierzbicka et al. 2021).

Recent advances in microbiome research have highlighted the multifaceted role of probiotics in modulating host immune responses and protecting against pathogenic infections. Probiotics, particularly *Lactobacillus* strains, exert beneficial effects through diverse mechanisms, including cytokine regulation, enhancement of innate and adaptive immunity, and reinforcement of epithelial barrier integrity. For example, Yeşilyurt et al. (2021) demonstrated that probiotics enhance cytokine production, increase immunoglobulin levels, stimulate mononuclear cell proliferation, and elevate natural killer cell activity, while also activating macrophages. Similarly, Begum et al. (2021) reported that probiotics modulate autoimmunity and strengthen host defences against bacterial and protozoal pathogens. Importantly, the concurrent use of multiple probiotic strains has been shown to produce synergistic immunological benefits, amplifying the overall protective response (J. Lee, Jo, et al. 2024).

Our findings demonstrate that probiotic–postbiotic supplementation effectively attenuated *E. coli*–induced renal and testicular damage in male Wistar rats. Treatment reduced pro‑inflammatory cytokine expression (TNF‑α, IL‑6), downregulated the pro‑apoptotic marker Bax, and upregulated the anti‑apoptotic gene Bcl‑2, thereby contributing to the restoration of tissue homeostasis. These results are consistent with previous reports; for example, Li et al. (2021) showed that pretreatment with *L. rhamnosus* protected against *E. coli*–induced mastitis by lowering TNF‑α and IL‑1β levels, highlighting the immunomodulatory potential of probiotics in infection models. Similarly, our observation of improved sperm quality and modulation of apoptotic gene expression parallels the findings of Keshtmand et al. (2025), who reported protective effects of *L. casei* and *Bifidobacterium lactis* on sperm parameters in diabetic rats. Taken together, these studies reinforce the translational relevance of probiotic–postbiotic interventions in mitigating infection‑associated oxidative stress, apoptosis, and reproductive dysfunction.

These findings are consistent with previously reported murine experimental models of *E. coli* infection, which similarly demonstrate marked inflammatory and apoptotic responses following bacterial challenge (Peng et al. 2024). Beyond their immunomodulatory activity, probiotics contribute to maintaining microbial balance within the male genitourinary tract, where their antimicrobial metabolites help limit pathogenic colonization and support tissue homeostasis.

In the context of the *E. coli* challenge model, the modulatory effects of probiotic and postbiotic supplementation are best interpreted through their capacity to regulate host inflammatory and apoptotic responses rather than through broad mechanistic pathways. Both interventions attenuated infection‑induced tissue injury by reducing pro‑apoptotic signalling and preserving epithelial integrity, consistent with murine models demonstrating similar protective effects against *E. coli*–mediated renal and reproductive damage (Vinayamohan et al. 2024; Hreha et al. 2026). This targeted modulation of host responses highlights the relevance of probiotic‑ and postbiotic‑derived metabolites in mitigating bacterial challenge–associated dysfunction.

Taken together, our study provides novel evidence that probiotic–postbiotic supplementation protects against *E. coli*–induced renal and testicular injury by enhancing antioxidant defences, modulating apoptosis, and suppressing inflammation. These findings reinforce the growing consensus that probiotics and postbiotics represent promising adjuncts or alternatives to antibiotics, particularly in the era of escalating resistance. Future research should focus on clinical translation, including dose optimization, strain selection, and long‐term safety evaluation, to fully harness the therapeutic potential of microbiota‐based interventions.

## 5 Conclusion

The present study demonstrates that supplementation with a probiotic–postbiotic mixture (*L. casei*, *L. rhamnosus*, and *L. helveticus*) exerts protective effects against *E. coli*–induced renal and testicular damage in male Wistar rats. These benefits appear to be mediated through multiple mechanisms, including enhancement of antioxidant defences, inhibition of toxic compound activity, scavenging of free radicals, and modulation of inflammatory and immune responses.

Our findings suggest that probiotics and postbiotics may serve as promising adjuncts or alternatives to conventional therapies for mitigating infection‐related tissue injury and potentially reducing the harmful impacts of environmental pollutants and pathogenic microorganisms. Nevertheless, further investigations are required to define the optimal strains, dosages, and timing of administration, as well as to evaluate long‐term safety and efficacy. Rigorous preclinical and clinical studies will be essential to translate these results into practical applications for human health and to establish microbiota‐based interventions as feasible candidates in the prevention and treatment of infection‐induced infertility and renal dysfunction.

## Author Contributions

**Saeid Sedghi** and **Zahra Keshtmand**: conceptualization. Saeid Sedghi, Zahra Keshtmand, and **Saba Hosseini**: methodology and software. Saeid Sedghi: data curation, writing – original draft preparation. Saeid Sedghi and Zahra Keshtmand: writing – reviewing and editing. All authors read and approved the final manuscript.

## Funding

The authors have nothing to report.

## Ethics Statement

Ethical approval was obtained under code IR.IAU.CTB.REC.1401.091.

## Conflicts of Interest

The authors declare conflicts of interest.

## Data Availability

The data used to support the findings of this study are included within the article.
